# Self‐concept and depressive symptoms three years after stroke: An evaluation of predictive value, the role of subdomains and individual importance

**DOI:** 10.1111/jnp.70005

**Published:** 2025-07-17

**Authors:** Simon Ladwig, Katja Werheid

**Affiliations:** ^1^ Clinical Neuropsychology and Psychotherapy, Faculty of Psychology and Sports Science Bielefeld University Bielefeld Germany

**Keywords:** depression, importance‐weighting, self‐concept, stroke

## Abstract

Depressive symptoms (DS) after stroke are associated with marked negative consequences for rehabilitation. Identifying determinants of DS is needed to enable prediction and develop psychological interventions. A promising candidate may be self‐concept and changes thereof, so‐called self‐discrepancy. Consulting recent self‐concept models, we investigated the role of self‐concept subdomains and their individual importance. Within a prospective longitudinal study, 120 stroke survivors were interviewed via telephone 3 years post‐ictus to assess present and past self‐concept, self‐discrepancy, self‐concept subdomains and DS. The association of self‐concept measures and DS was investigated using an ANCOVA. Controlling for established determinants (age, sex, history of depression, functional independence, social support), multiple regression analyses were used to examine the independent influence of self‐concept measures and the role of subdomains and importance‐weightings. Self‐discrepancy showed a significant interaction with DS (*F* (1, 118) = 32.69, *p* < .001, *η* ^2^ = .22). DS showed a stronger association with present (*r* = −.72) than with past self‐concept (*r* = −.34) and self‐discrepancy (*r* = −.47; all *p* < .001). Age, history of depression, social support and present self‐concept were independent predictors of DS while functional independence was not (∆*F* (1, 113) = 48.04, *p* < .001). Importance‐weighting of subdomains did not affect explained variance, though the number of self‐concept subdomains showing significant association with DS increased. Findings propose appraisals of self‐concept as independent predictors of DS after stroke. Considering individual importance of subdomains reveals their differential influence. The results suggest investigating the use of general self‐concept for prediction and considering the individual relevance of subdomains in psychological interventions after stroke.

## INTRODUCTION

Depression is the most common mental disorder post‐stroke, occurring in a third of all survivors (Liu et al., 2023). Individuals with increased depressive symptoms (DS) face increased adversities like reduced quality of life, cognition, functional independence and rehabilitation efficiency as well as increased mortality compared to stroke survivors with sub‐threshold DS (Blöchl et al., 2019; Cai et al., 2019; Gillen et al., 2001; Kim et al., 2018; Terroni et al., 2012). Hence, numerous studies have aimed to identify predictors of DS to increase knowledge about their pathogenesis, enable prediction and improve treatment use. Depression after stroke is reported to be vastly undertreated while the meta‐analytic evidence of treatment efficacy still has very low certainty (Allida et al., 2023; Ladwig et al., 2018).

Meta‐analyses report history of depression, stroke severity, functional dependence, cognitive status and social support as the most consistently proven predictors of DS (Ayerbe et al., 2013; de Ryck et al., 2014; Kutlubaev & Hackett, 2014). A recent study aimed to validate these five predictors in multivariable analyses using data of two distinct longitudinal samples, and confirmed history of depression, functional dependence and social support as independent predictors (Ladwig et al., 2023). While all three variables may be used in prediction and early identification, social support may be the only entity amenable to therapeutic interventions. Ladwig et al. (2023) also showed that intraindividual changes in functional dependence and social support explained variance in DS in addition to the established risk factors, which were assessed as status variables at distinct measurement points. The findings support that the dynamics of determinants, which may reflect the subjective experience of stroke more adequately than status measures, may play a crucial role in the pathogenesis of DS (Jørgensen et al., 1995; Kreisel et al., 2007; Palmer & Glass, 2003; Thompson & Ryan, 2008; Volz et al., 2016).

The experience of brain injury is often characterized by a changed or lost sense of self (Ellis‐Hill & Horn, 2000; Lapadatu & Morris, 2019; Ownsworth, 2014). Changes in self‐concept, so‐called self‐discrepancy, are repeatedly demonstrated and show strong associations with mental health after brain injury in general (e.g. Beadle et al., 2016; for an overview: Ownsworth, 2014) and stroke in particular (Ellis‐Hill & Horn, 2000; Lapadatu & Morris, 2019; Secrest & Zeller, 2007).

Self‐concept is defined as a cognitive description of the self (e.g. ‘impatient’ or ‘patient’) in distinction to self‐esteem, which represents the evaluation of the self‐concept (e.g. ‘good’ or ‘bad’; Ownsworth, 2014). Self‐concept may be a global appraisal of one's self or refer to specific domains, which is synthesized in the multidimensional and hierarchical model of self‐concept. Shavelson et al. (1976) proposed that the global self‐concept consists of the self‐concept domains of social, emotional, physical and academic self‐concept, which themselves consist of subdomains, for example peers and significant others for the social domain.

Self‐discrepancy is usually assessed by individuals' retrospective ratings of characteristics before and after the brain injury and the global difference scores between these two measurements. Secrest and Zeller (2007) published the so far only longitudinal study including a stroke sample which investigated self‐concept‐related constructs in 33 individuals one and six months post‐ictus. Using a self‐developed scale (Secrest & Zeller, 2006), the authors aimed to assess continuity and discontinuity of the self. At both measurement times, continuity of the self and discontinuity showed strong bivariate associations with DS. However, the sample size was limited, the applied scale was never validated with established measures of self‐discrepancy and only bivariate associations were investigated. Hence, the results should be viewed with caution and it is unclear to what extent the reported associations were independent of the other examined variables as no multivariable analyses were conducted. This is essential in the multifactorial pathogenesis of DS after stroke (Ladwig et al., 2023).

These results were extended by Lapadatu and Morris (2019), who investigated present self‐concept (i.e. after stroke) and self‐discrepancy in 65 stroke survivors using the Head Injury Semantic Differential Scale–III (HISDS‐III; Carroll & Coetzer, 2011; Tyerman & Humphrey, 1984), which is the most widely used self‐discrepancy measure after brain injury (Ownsworth, 2014). Participants rated their present self‐concept as more negative than their past self‐concept (i.e. before stroke), which was associated with lower self‐esteem and quality of life as well as higher anxiety and depression severity. Notably, they found that present self‐concept was more strongly associated with DS and quality of life than self‐discrepancy. In a multiple regression on depression including both variables, self‐discrepancy was even excluded from the model. Moreover, they compared past and present self‐concept ratings to population norms from the literature (Wright & Telford, 1996), which showed a higher difference between the norm and past self‐concept than between the norm and present self‐concept. Therefore, the authors concluded that self‐discrepancy is based on an idealization of the past self rather than a devaluation of the present self. This implies an additional association of depression with past self‐concept, which was, however, not directly investigated.

The Lapadatu and Morris (2019) study left open several issues. First, the time since stroke in their sample ranged from one to 15 years (*M* = 5.6 years). Thus, there was considerable heterogeneity with respect to time‐dependent experiences in the course of stroke rehabilitation, for example returning home after inpatient treatment or returning to work. The assertion that determinants of DS and the subjective experience of stroke may change over time (Herrmann & Wallesch, 1993; Werheid, 2016) could therefore not be addressed. Also, Lapadatu and Morris (2019) used the Hospital Anxiety and Depression Scale to assess DS, which was developed for the inpatient setting and has proven to be of limited diagnostic use in stroke populations (Meader et al., 2013). These points call for a reexamination of these findings, addressing all three reference times (present, past and discrepancy) within a more constrained time frame and using depression scales that are suitable for outpatient examination.

Global self‐concept changes show consistent associations with mental health after stroke (Ellis‐Hill & Horn, 2000; Lapadatu & Morris, 2019; Secrest & Zeller, 2007), yet the individual importance of self‐concept domains and subdomains may differ markedly between individuals. Most of the mentioned studies apply a version of the HISDS (Tyerman & Humphrey, 1984) to assess self‐concept. This scale contains 18 items requiring a rating between bipolar adjective pairs, for example unattractive‐attractive or withdrawn‐talkative. Considering these examples, it seems self‐evident that individuals attribute different importance to these characteristics for their global self‐concept. For example, a person may value talkativeness higher than their attractiveness and therefore may be more strongly affected by subjective changes in the former after stroke. This concept already dates back to William James (1890/1950) and was later labelled as the individually importance‐weighted model (IIWM) of self‐esteem, which proposes that the interaction of individual *ratings* in self‐concept domains with the individual *importance* of each domain predicts self‐esteem more adequately than equally weighted ratings of self‐concept domains alone (Marsh & Scalas, 2018). While definitions refer to self‐concept as a description of one's self and self‐esteem as its evaluation (Ownsworth, 2014), the differentiation of these two is sometimes vague in research (Marsh & Scalas, 2018; Scalas et al., 2017). This may be based on the ratings of self‐concept domains often incorporating some kind of implicit evaluation, for example unattractive versus attractive.

The IIWM is often referenced in self‐concept literature (Mruk, 2006; Schacter et al., 2011). However, empirical evidence does not support the IIWM for *global self‐esteem* (Scalas et al., 2013). However, its validity was postulated and demonstrated for narrower self‐concept domains like music self‐concept, which are only important to a few people (Marsh, 1986; Scalas et al., 2017), and this may apply to the experience of stroke survivors as well. A stroke may specifically impair domains that are of high importance to an individual, but not as important to others, for example talkativeness (Lai et al., 2002). Moreover, patterns of impairment vary considerably among survivors. This interaction of domain importance and impairment enables both a high diversity and uniqueness of stroke experiences. Therefore, the individual stroke‐specific changes in self‐concept may resemble the processes involved in self‐concept formation for narrow domains rather than global self‐concept. This would argue for the investigation of individual importance‐weightings of self‐concept domains after stroke in accordance with the IIWM (Scalas et al., 2017).

This study pursues two objectives. First, we intend to confirm and extend Lapadatu and Morris' (2019) findings on the association of self‐concept and DS after stroke, using refined methodology and controlling for established risk factors (Ladwig et al., 2023). Second, we aim to apply the assumptions of the IIWM to self‐concept in stroke survivors by investigating the effects of individual importance of subdomains (Marsh & Scalas, 2018; Scalas et al., 2017). This may increase the understanding of self‐concept changes post‐stroke and enable the development of tailored interventions.

## MATERIALS AND METHODS

**FIGURE 1:**
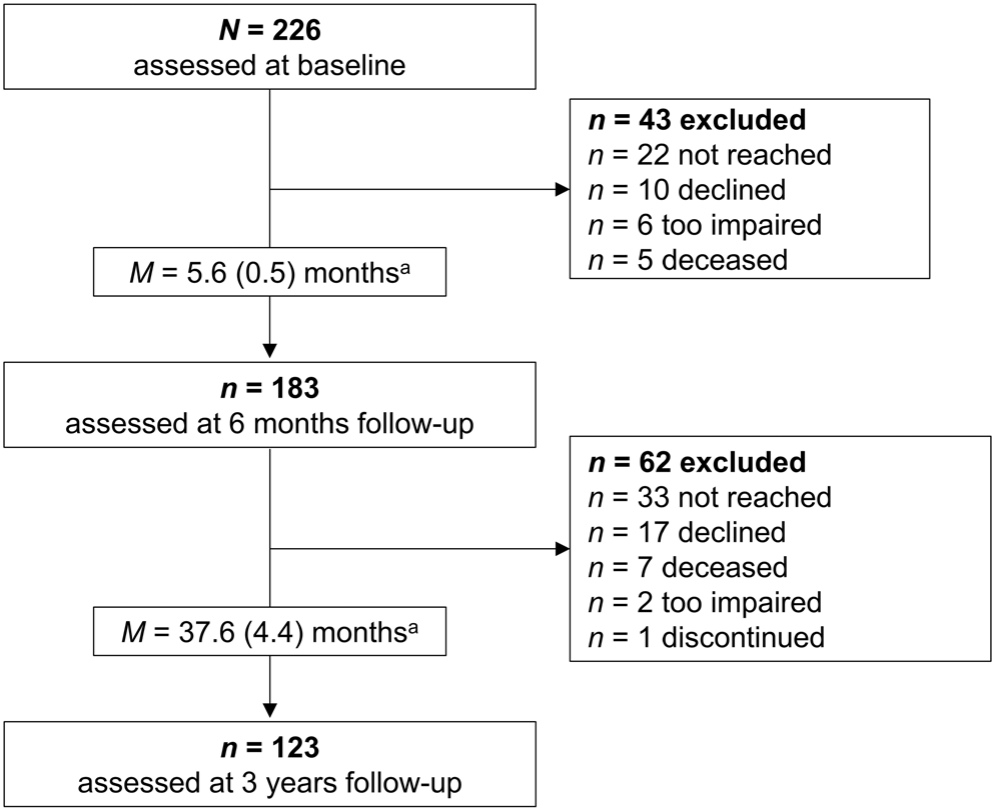
Flow chart of study sample. HISDS, Head Injury Semantic Differential Scale. ^a^Time since baseline assessment. Value in brackets = standard deviation. ‘Too impaired’ refers to report of caregivers or participants.

As part of the prospective longitudinal study PoStDAM (Post‐Stroke Depression: Early Assessment for improved Management), follow‐up telephone interviews were conducted between April and October 2022, 3 years after stroke. The PoStDAM study was previously described in detail (Ladwig et al., 2022). The study consecutively recruited people with ischemic stroke, sufficient language comprehension (Aphasia Screening Test comprehension score ≥ 11 in age ≤ 70 years and a score ≥ 10 in age ≥ 71 years, German version; Kroker, 2006) and sufficient cognition (MMSE score ≥ 18, German ‘Hogrefe’ version; Folstein et al., 1975; Kaiser et al., 2009) from a stroke unit. Eligibility criteria were chosen to assure validity of self‐report outcomes and align the sample to a previous study, which shared a main research question with the PoStDAM study (Hirt et al., 2020). Participants were excluded if they had another impairing or terminal disease (e.g. cancer, neurodegenerative diseases or acute life‐threatening conditions). After giving written informed consent, participants were assessed on the stroke unit and in a follow‐up telephone interview 6 months later where they were also asked for their consent to be contacted in the future. Consenting participants were informed by post about the three‐year follow‐up, received updated information on data privacy and were contacted again via telephone to give verbal informed consent. The baseline and the first follow‐up were approved by the ethics commission at Universität Potsdam (Application 5/2018). The second follow‐up was approved by the ethics commission at the department of psychology, Humboldt‐Universität zu Berlin (Application 2022‐12R1). Participant flow and reasons for attrition are described in Figure 1. The STROBE cross‐sectional checklist was used when writing this report (von Elm et al., 2007).

At baseline, examiners (neuropsychologist or trained and consistently supervised psychology graduate students) evaluated the inclusion and exclusion criteria, and assessed demographics, history of depression (self‐report: yes/no), DS (Patient Health Questionnaire‐9 (PHQ‐9), German version; Löwe et al., 2002) and perceived social support (Social Support Questionnaire (FSozU K‐14); Fydrich et al., 2009). The PHQ‐9 (range: 0–27) was chosen as it allows a severity rating of the nine depression criteria according to DSM‐5 (American Psychiatric Association, 2013). The German version shows a sensitivity of 95% and a specificity of 86% to detect major depression in the general population (Gräfe et al., 2004). The PHQ‐9 was also reported as one of the most accurate depression assessments in stroke populations in a meta‐analysis including data of almost 3000 participants (Meader et al., 2013). The FSozU K‐14 is a short version of a well‐validated German social support scale. In a sample of 2507 participants, Fydrich et al. (2009) demonstrated its convergent and discriminant validity. Neurologists rated stroke severity with the modified National Institutes of Health Stroke Scale (mNIHSS, range = 0–31, German version; Berger et al., 1999; Meyer et al., 2002) and nurses rated functional independence with the Barthel Index (range = 0–100, German version; Heuschmann et al., 2005). Higher scores indicate higher severity of DS or stroke, higher perceived social support and higher independence, respectively. At follow‐up, participants were asked to complete a German version of the HISDS‐III, which consists of 18 items consisting of bipolar adjective pairs (Table 3) with a 7‐point Likert scale. Since its first publication, the HISDS experienced several updates (Tyerman & Humphrey, 1984). Based on the second version (HISDS‐II; Ellis‐Hill & Horn, 2000), two independent German translations were published (Doering et al., 2010; Hämmerling et al., 2008), where only Doering et al. (2010, 2011) described the process of translation and demonstrated criterion and construct validity. The most recent HISDS‐III (Carroll & Coetzer, 2011) omitted some items and reworded items to improve validity, which showed good reliability and criterion validity in people with traumatic brain injury. We applied these adjustments and derived the German HISDS‐III. In this study, participants first rated their past self *within six months before the stroke*. For individual importance ratings, they rated the order of the three most important subdomains (=items) at that time. The ratings of the items and the three most important subdomains were repeated for their present self *within the last six months*. Past and present self‐concept are measured by the respective sum scores of the items, which may range from 18 to 126, with higher values indicating a more positive self‐concept. In this sample, reliability for both past and present HISDS‐III was excellent (Cronbach's *α*_past_ = .94; *α*_present_ = .96). In accordance with previous studies (Lapadatu & Morris, 2019), self‐discrepancy was calculated by subtracting past self‐concept from present self‐concept. Hence, negative values indicate decreasing self‐concepts over time. In addition, participants completed the Barthel Index (functional independence), the PHQ‐9 (DS) and the FSozU K‐14 (social support) at the three‐year follow‐up.

We conducted a repeated‐measures ANOVA using the HISDS‐III scores as the dependent variable and the reference times (past vs. present) as the within‐subjects factor. To the same analysis, DS (PHQ‐9) was included as a covariate (ANCOVA). In addition, we calculated Pearson correlations with Bootstrapping (1000 samples) of past and present self‐concept and self‐discrepancy with DS. Correlation coefficients were compared based on 95% confidence intervals. We hypothesized the effect sizes to resemble the pattern found by Lapadatu and Morris (2019) with present self‐concept showing the strongest, discrepancy the second strongest and past self‐concept the third strongest association with DS. The additional predictive value of self‐concept/‐discrepancy was investigated by a stepwise multiple regression analysis (change in *R* ^2^) predicting the PHQ‐9 sum score and including age, sex, history of depression, functional independence and social support as predictors in the first step. It may be noted that the demographics age and sex do not show consistent associations with DS after stroke, yet they represent relevant confounders which are often controlled for (Ayerbe et al., 2013; de Ryck et al., 2014; Kutlubaev & Hackett, 2014). Present self‐concept was added in the second step, self‐discrepancy in the third step and past self‐concept in the fourth step. Changes in explained variance (*R* ^2^) were calculated and tested for significance using ANOVAs. Kolmogorov–Smirnov tests showed that the scores of PHQ‐9 and HISDS measures (present, past, discrepancy) were not normally distributed (all *p* < .001). However, ANOVA and linear regression analyses are robust to violations of the normality assumption (Schmider et al., 2010; Schmidt & Finan, 2018) and Pearson correlation was adjusted by bootstrapping.

For our second research question of investigating the IIWM assumptions in self‐concept after stroke, a multiple regression analysis predicting the PHQ‐9 was conducted including all 18 item scores of the *present HISDS‐III* as predictors (*subdomain content* model). To consider the individual importance of subdomains (*subdomain content X importance* model), this analysis was repeated with weighted item scores of the *present HISDS‐III*. Each item was weighted according to the place it received in the importance rating: first = factor 4, second = factor 3, third = factor 2 and the remaining = factor 1. For example, if an individual rated item 2 with a score of 5 and rated item 2 as the most important subdomain (first place), the weighted score equals 20 (5*4). Finally, to consider only the importance of ratings independent from the items' content (*importance* model), three variables were transformed (first place, second place, third place), which consisted of the present HISDS‐III scores that participants rated on the most, second most and third most important item. For example, if a person rated the item ‘unhappy‐happy’ with a score of 5 and this item also as the most important subdomain (first place), the value of this person in the variable ‘first place’ equals 5. The first two models on the subdomain effects (*subdomain content* and *subdomain content X importance*) were compared based on the significance of predictors and explained variance (adjusted *R* ^2^). The third model (*importance*) was compared with the other two models based on explained variance (adjusted *R* ^2^). Case‐wise deletion was applied for missing data. For drop‐out analysis, individuals participating at the three years follow‐up were compared with non‐participating ones using χ^2^‐tests for categorical and independent *t*‐tests for continuous variables applying a Bonferroni‐adjusted significance level. All analyses were conducted with SPSS, version 28 (IBM, 2021).

We report the power analysis with G‐Power (version 3.1.9.7; Faul et al., 2009) for the analysis requiring the highest sample size. For the regression model of the subdomain analysis, choosing an effect size of .20 (*f* ^2^), a power of .80 and 18 predictors, the power analysis resulted in a required sample size of *N* = 116. Original data are available online (Ladwig, 2024).

## RESULTS

At baseline, *N* = 226 stroke survivors were recruited and *N* = 123 participants were assessed after 3 years. Three participants were excluded from analysis due to missing data in the HISDS‐III. Hence, data of *N* = 120 individuals were used for the main analyses. Descriptive statistics of demographic, clinical and self‐concept measures, and results of drop‐out analyses are shown in Table 1. After 3 years, 18.3% (*n* = 22) participants reported depressive symptoms above the cut‐off (PHQ‐9 ≥ 10; Meader et al., 2013). Stroke survivors participating after 3 years were significantly younger, and had higher cognition and higher functional independence at baseline than those who did not participate. There was no selective attrition regarding sex, education, stroke characteristics (location, severity), social support or DS.

**TABLE 1:** Descriptive statistics and drop‐out analysis for study sample at baseline and 3‐year follow‐up.

	Baseline (*N* = 223)^a^	3‐year follow‐up (*N* = 120)^a^	Drop‐out^†^	
	*N* (%)	*N* (%)	χ^2^	*p*
Sex (female)	107 (48.0)	57 (47.5)	.02	.876
History of depression (yes)	59 (26.5)	35 (29.2)	.98	.322
First‐ever stroke (yes)	176 (78.9)	95 (79.2)	.01	.924
Lesion location				
Right hemisphere	87 (39.0)^b^	44 (36.7)^c^	.94	.624
Left hemisphere	93 (41.7)^b^	51 (42.5)^c^		
Other^‡^	28 (12.6)^b^	17 (14.2)^c^		
Antidepressant medication (yes)	24 (10.8)	11 (9.2)^d^	.69	.407

	*M*	SD	Range	*M*	SD	Range	*t*	*p*
Age (years)	70.7	12.8	24/97	68.3	13.0	24/92	**3.00**	.**003**
Education (years)	13.8	3.6	6/23	14.1	3.2	6/22	−1.38	.167
Cognition (MMSE)	26.3	2.8	18/30	27.1	2.2	19/30	**−4.50**	**<.001**
Stroke severity (mNIHSS)^e^	2.5	3.1	0/27	2.1	2.2	0/11	2.31	.022
Functional independence (Barthel Index)	74.0	28.6	0/100	90.8	15.2	15/100	**−4.91**	**<.001**
Social support (FSozU K‐14)	4.4	.6	2.1/5.0	4.2	.7	1.9/5.0	−.31	.761
Depressive symptoms (PHQ‐9)	5.6	4.7	0/27	5.4	5.2	0/27	−.49	.622
Past self‐concept (HISDS‐III_past_)	–	–	–	101.5	16.7	33/126	–	–
Present self‐concept (HISDS‐III_present_)	–	–	–	93.7	21.0	27/126	–	–
Self‐discrepancy (HISDS‐III_present‐past_)	–	–	–	−7.8	20.0	−89/35	–	–

*Note*: Significant *p*‐values are marked as bold. Data of ^a^ *n* = 3 participants were excluded due to missing HISDS‐III data, ^b^ *n* = 209, ^c^ *n* = 113, ^d^ *n* = 119, ^e^ *n* = 222 participants available.

Abbreviations: FSozU K‐14, Social Support Questionnaire, 14 item version; HISDS‐III, Head Injury Semantic Differential Scale–III; MMSE, Mini‐Mental State Examination; mNIHSS, modified National Institutes of Health Stroke Scale; PHQ‐9, Patient Health Questionnaire‐9.

^†^ Bonferroni‐adjusted significance level of *p* = .004 was applied.

^‡^ Bilateral and/or subcortical location.

### Self‐discrepancy after stroke

**FIGURE 2:**
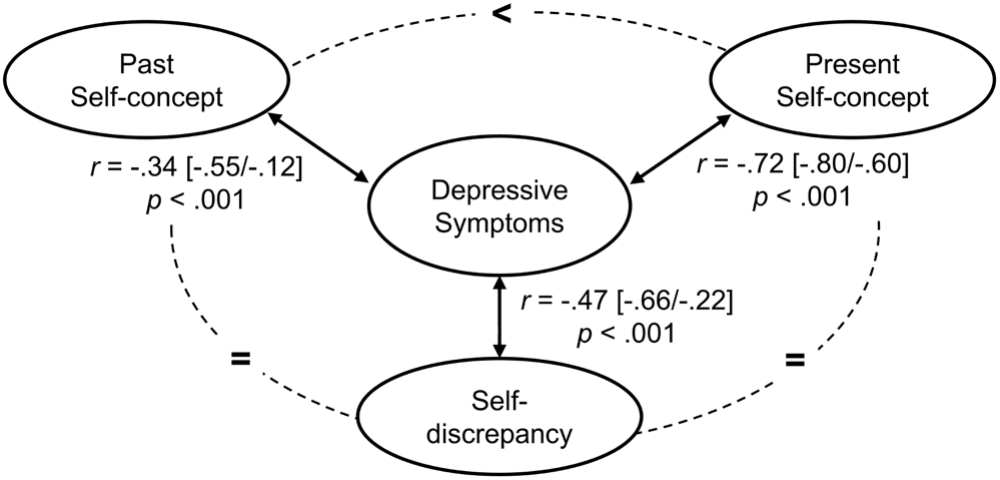
Pearson correlation of depressive symptoms with self‐concept measures based on bootstrapping with 1000 samples. Values in squared brackets = limits of 95% confidence intervals. The dashed line and relational operators indicate significant (<) and non‐significant differences (=) between coefficients.

The repeated‐measures ANOVA showed a significant main effect of the within‐subjects factor reference time, *F* (1, 120) = 19.23, *p* < .001, *η* ^2^_p_ = .14. Participants rated their past self‐concept more positively than their present self‐concept (see Table 1). In the ANCOVA including DS as a covariate, the main effect of the within‐subjects factor reference time was no longer significant, *F* (1, 118) = .64, *p* = .426, *η* ^2^_p_ = .01. Yet, the interaction effect of the within‐subjects factor and the covariate was significant, *F* (1, 118) = 32.69, *p* < .001, *η* ^2^_p_ = .22. The main effect of the covariate was also significant, *F* (1, 118) = 82.97, *p* < .001, *η* ^2^_p_ = .41. Figure 2 displays the correlations of DS with past and present self‐concept and self‐discrepancy. Based on the confidence intervals, the association of DS with present self‐concept was significantly higher than the association with past self‐concept.

### Self‐concept as a determinant of post‐stroke depressive symptoms

Results of the stepwise multiple regression analysis predicting DS are displayed in Table 2. In the first step, younger age, history of depression, lower functional independence and lower social support predicted DS significantly, explaining 45% of the variance, ∆*F* (5, 114) = 20.63, *p* < .001. In the second step, including present self‐concept, younger age and lower social support remained significant predictors. Negative present self‐concept was a significant predictor as well, while history of depression and functional independence did not reach significance. Explained variance significantly increased to 61%, ∆*F* (1, 113) = 48.04, *p* < .001. In the third step, including self‐discrepancy, younger age, history of depression, lower social support and negative present self‐concept were significant predictors. Functional independence and self‐discrepancy did not reach significance. Explained variance remained at 61%, ∆*F* (1, 112) = .64, *p* = .426. The fourth step including past self‐concept was not conducted as the predictor was excluded from analysis.

**TABLE 2:** Results of stepwise multiple linear regression analysis predicting depressive symptoms (PHQ‐9).

	Step 1			Step 2			Step 3		
	*β*	95%CI	*p*	*β*	95%CI	*p*	*β*	95%CI	*p*
Age	**−.26**	**−.41/−.12**	**<.001**	**−.20**	**−.33/−.08**	.**002**	**−.21**	**−.33/−.07**	.**002**
Sex	−.05	−.19/.09	.445	−.11	−.23/.01	.059	−.11	−.23/.01	.065
History of depression	.**21**	.**07/.35**	.**004**	.12	−.01/.24	.055	.**13**	.**01/.25**	.**043**
Functional independence (Barthel Index)	**−.32**	**−.47/−.17**	**<.001**	−.13	−.27/.01	.076	−.12	−.26/.02	.084
Social support (FSozU K‐14)	**−.44**	**−.57/−.29**	**<.001**	**−.23**	**−.36/−.09**	**<.001**	**−.24**	**−.37/−.10**	**<.001**
Present self‐concept (HISDS‐III_present_)	–	–	–	**−.51**	**−.67/−.37**	**<.001**	**−.47**	**−.66/−.28**	**<.001**
Self‐discrepancy (HISDS‐III_present‐past_)	–	–	–	–	–	–	−.06	−.22/.10	.426
	*R* ^2^ = .45			*R* ^2^ = .61, ∆*R* ^2^ = .157, *p* < .001			*R* ^2^ = .61, ∆*R* ^2^ = .002, *p* = .42		

*Note*: Bold values are significant at *p* < .05.

Abbreviations: CI, confidence interval; FSozU K‐14, Social Support Questionnaire, 14 item version; HISDS‐III, Head Injury Semantic Differential Scale–III; PHQ‐9, Patient Health Questionnaire‐9; *R* ^2^, adjusted *R* ^2^; *β*, standardized regression coefficient.

### IIWM assumptions in self‐concept after stroke

For the second objective, investigating the assumptions of the IIWM to self‐concept after stroke, two regression models using the present HISDS‐III were calculated: one model with all 18 unweighted items (*subdomain content* model) and one model with all 18 importance‐weighted items (*subdomain content X importance* model). Table 3 displays the frequencies of the relevance weightings and the individual results of the regression analyses. The unweighted regression model explained 51% of the explained variance (∆*F* (18, 101) = 7.88, *p* < .001), and only item 16 (‘Talkative’) was a significant predictor with lower scores predicting DS (*p* < .023). Using the importance‐weighted scores, explained variance remained about the same with 48% (∆*F* (18, 100) = 6.96, *p* < .001), yet six items were significant predictors: Item 1 (‘Interested’), item 2 (‘Happy’), item 8 (‘Self‐confident’), item 13 (‘Capable’), item 14 (‘Independent’) and item 15 (‘Active’) (*p* = .005–.042). More negative ratings on these subdomains were associated with higher severity of DS (*β* = −.20 to −.14).

**TABLE 3:** Distribution of importance‐weightings for present HISDS‐III items (first to third place) and results of multiple linear regression analyses using the unweighted and importance‐weighted item scores to predict depressive symptoms (PHQ‐9; *N* = 120).

Item	Poles	1st place	2nd place	3rd place	Unweighted			Importance‐weighted		
		*N* (%)			*β*	95%CI	*p*	*β*	95%CI	*p*
1	Bored – Interested	20 (16.7%)	10 (8.3%)	9 (3.9%)	.02	−.19/.23	.832	**−.14**	**−.28/−.01**	.**042**
2	Unhappy – Happy	15 (12.5%)	11 (9.2%)	7 (5.8%)	−.11	−.37/.15	.404	**−.20**	**−.35/−.05**	.**009**
3	Helpless – In control	11 (9.2%)	4 (3.3%)	3 (2.5%)	−.03	−.34/.29	.866	−.10	−.25/.06	.226
4	Worried – Relaxed	2 (1.7%)	5 (4.2%)	4 (3.3%)	.10	−.15/.36	.423	−.09	−.23/.06	.256
5	Dissatisfied – Satisfied	12 (10.0%)	16 (13.3%)	12 (10.0%)	−.15	−.44/.12	.271	−.12	−.27/.02	.097
6	Unattractive – Attractive	0 (0%)	4 (3.3%)	0 (0%)	.06	−.16/.28	.588	−.02	−.18/.14	.855
7	Despondent – Hopeful	9 (7.5%)	9 (7.5%)	7 (5.8%)	−.05	−.31/.21	.696	−.11	−.26/.03	.131
8	Lacks confidence – Self‐confident	5 (4.2%)	6 (5.0%)	8 (6.7%)	−.10	−.39/.19	.504	**−.20**	**−.36/−.04**	.**015**
9	Emotional – Stable	4 (3.3%)	6 (5.0%)	13 (10.8%)	−.17	−.43/.09	.202	−.12	−.26/.02	.094
10	Worthless – Of value	0 (0%)	3 (2.5%)	6 (5.0%)	−.21	−.47/.05	.107	−.12	−.27/.04	.132
11	Aggressive – Unaggressive	1 (.8%)	4 (3.3%)	4 (3.3%)	−.06	−.26/.14	.546	−.13	−.27/.01	.074
12	Irritable – Calm	4 (3.3%)	8 (6.7%)	5 (4.2%)	−.06	−.30/.19	.643	−.09	−.24/.05	.204
13	Uncapable – Capable	0 (0%)	4 (3.3%)	2 (1.7%)	.19	−.07/.45	.148	**−.18**	**−.33/−.02**	.**030**
14	Dependent – Independent	11 (4.8%)	8 (6.7%)	12 (10.0%)	−.02	−.25/.21	.887	**−.15**	**−.29/−.01**	.**037**
15	Inactive – Active	9 (7.5%)	7 (5.8%)	6 (5.0%)	−.20	−.45/.05	.111	**−.20**	**−.33/−.06**	.**005**
16	Withdrawn – Talkative	5 (4.2%)	4 (3.3%)	4 (3.3%)	**−.27**	**−.51/−.04**	.**023**	−.04	−.19/.12	.623
17	Unfriendly – Friendly	4 (3.3%)	6 (5.0%)	13 (10.8%)	.09	−.09/.27	.315	−.14	−.29/.01	.054
18	Impatient – Patient	8 (6.7%)	5 (4.2%)	5 (4.2%)	.10	−.12/.33	.356	−.07	−.22/.07	.319
					*R* ^2^ = .51			*R* ^2^ = .48		

*Note*: Annotation: The English item labels according to Tyerman and Humphrey (1984) and Reddy et al. (2017). Bold values are significant at *p* < .05.

Abbreviations: CI, confidence interval; HISDS‐III, Head Injury Semantic Differential Scale–III; PHQ‐9, Patient Health Questionnaire‐9; *β*, standardized regression coefficient.

In addition, a regression model was calculated using the individual scores of the items rated as first, second and third most important, independent from item content (*importance* model). The model explained 45% of variance in DS (∆*F* (3, 115) = 32.51, *p* < .001). The most relevant subdomain fell just short of the significance level (*β* = −.20, 95%CI = −.40/.01, *p* = .06). The second (*β* = −.32, 95%CI = −.54/−.11, *p* < .01) and third most important subdomain (*β* = −.25, 95%CI = −.44/−.05, *p* < .05) were significant predictors.

## DISCUSSION

This study confirms the findings on the close relationship between self‐concept and self‐discrepancy with DS after stroke while overcoming previous methodological limitations (Lapadatu & Morris, 2019; Secrest & Zeller, 2007). We investigated the associations of all three relevant self‐concept measures (present, past and discrepancy) at a clearly determined time point 3 years after stroke and applied the PHQ‐9 to assess DS, a measure of proven diagnostic accuracy after stroke (Meader et al., 2013). Moreover, the results demonstrated the additional value of self‐concept in the prediction of DS after stroke. In our data, the effect even surpassed functional independence three years post‐stroke as the most consistently proven risk factor. Considering individual relevance of subdomains, as proposed by models of global self‐concept formation (Marsh & Scalas, 2018; Scalas et al., 2017), revealed associations of single subdomains with DS, which were concealed in the unweighted model. This finding suggests the assessment of subdomain relevance, which may allow for a more fine‐grained understanding of psychological stroke sequelae.

In accordance with previous findings, global self‐concept post‐stroke was more negatively rated than pre‐stroke (Ellis‐Hill & Horn, 2000; Lapadatu & Morris, 2019; Secrest & Zeller, 2007). The ANCOVA results indicate a relevant association of self‐discrepancy with DS after stroke. However, findings from a population‐based study suggested that self‐discrepancy as a subjective deterioration of self‐concept from past to present is also present in depression without brain injury (Sokol & Serper, 2017). Hence, depressed individuals seemed to rate their past self as more positive independently from brain injury. An additional amplification of self‐discrepancy after brain injury may be suspected, yet further research needs to examine if and how self‐discrepancy differs between individuals with and without brain injury. Also, future self‐concept after brain injury may be investigated as previous findings indicate that survivors wish to return to their pre‐illness state (Cusack, 2020; Tyerman & Humphrey, 1984; Wright & Telford, 1996).

Correlation analyses supported that present self‐concept was more strongly associated with DS than self‐discrepancy and past self‐concept, which replicated the pattern reported by Lapadatu and Morris (2019). This may be based on the negative perception bias in depression affecting self‐concept ratings. However, the perception of self‐discrepancy may also facilitate the development of depression, which again may promote a negative perception bias. This poses a chicken‐and‐egg problem, which cannot be resolved in a cross‐sectional design.

While we hypothesized the subjective changes after stroke to be crucial in the development of DS (Ladwig et al., 2023), self‐discrepancy added no significant predictive value to the regression model already including present self‐concept, which is in line with Lapadatu and Morris' (2019) findings. This suggests to limit assessments to present global self‐concept in the context of prediction for the sake of parsimony (Lapadatu & Morris, 2019).

Stepwise regression analyses confirmed the history of depression and social support as independent predictors of post‐stroke DS (Ladwig et al., 2023) while present self‐concept was demonstrated as a novel independent predictor, which significantly increased explained variance by 16%. Interestingly, the influence of functional independence ceased when present self‐concept was included in the model, which adds to previous findings reporting stronger associations of DS with self‐concept than with functional independence after stroke (Lapadatu & Morris, 2019). This is noteworthy because functional independence, regularly assessed by the Barthel Index, is the most consistently proven predictor of DS (Ayerbe et al., 2013; de Ryck et al., 2014; Kutlubaev & Hackett, 2014). This may be based on two aspects. Firstly, the HISDS‐III as the measure of self‐concept comprises items on independence, capability and activity (cf. Table 3) showing a content overlap with the Barthel Index. Secondly, subjective impairment is more relevant to mood than objective impairment (e.g. Duits et al., 2008). The Barthel Index is usually assessed by health professionals' external reports (Mahoney & Barthel, 1965). In this study, the Barthel Index was self‐rated by the survivors via telephone, which, while shown to be reliable (Della Pietra et al., 2011), may add subjectivity to the assessment. However, the HISDS‐III is inherently more subjective, entailing a self‐rating of the self. The subjective global evaluations of independence in the HISDS‐III may be more closely related to stroke survivors' mood than the more objective rating of independence in the Barthel Index. This is supported by recent findings on illness perception and quality of life after stroke (Ladwig et al., 2025; Roberts et al., 2024). Yet, methodological limitations must be noted. The mean independence was high in this sample 3 years after stroke, and variability was lower than in the baseline assessment (Table 1). Therefore, the insignificant effect between functional independence and DS may also be based on limited variance and ceiling values in this study. Yet, it should be noted that Lapadatu and Morris (2019) found self‐concept to be more strongly associated than functional independence and the investigated sample showed higher mean dependence (*M* = 77.3), actually comparable to our baseline sample. Moreover, the cross‐sectional design does not allow us to conclude a direction of the effect, that is depressive symptoms may also have biased the rating of the self‐reported determinants.

For this study's second objective, we investigated the influence of self‐concept subdomains and their interaction with individual importance rating as their influence is suggested by the IIWM (Marsh & Scalas, 2018; Scalas et al., 2013). To our knowledge, this is the first study to incorporate weighting assumptions of self‐concept models into the investigation of populations with brain injury. In the unweighted model, which did not consider individual importance, only the subdomain ‘talkativeness’ independently predicted DS. By contrast, when weighting the subdomains by their individual importance, six subdomains independently predicted DS, although explained variance remained similar to the unweighted model. Hence, considering the individual importance of self‐concept subdomains does not seem to improve the prediction of DS, which calls for using global evaluations of self‐concept in this regard. Risk prediction scales were developed for use in the acute phase to predict post‐stroke DS several months later and show adequate accuracy to rule out cases (Hirt et al., 2020; Ladwig et al., 2022). Yet, they require improvement in their rule‐in utility (Mitchell, 2011), which may be achieved by including economic measures of self‐concept. A promising candidate in this matter may be the other in the self‐scale (Heckmann et al., 2025). This scale is not only economic being a single‐item measure but also addresses cognitive and verbal stroke impairments by applying a visualization aid.

The results of the importance‐weighted regression model may be interpreted to be in line with the IIWM's assumptions (Marsh & Scalas, 2018). We hypothesized the formation of global self‐concept after stroke and brain injury to resemble the formation of specific self‐concepts, where the IIWM was shown to be valid (Scalas et al., 2017). This may be based on the specific consequences of stroke sequalae for activities and participation, which may differ markedly among survivors (Lai et al., 2002). On the contrary, the regression model, which only considered the three most important subdomain scores independently from subdomain content, showed a similar level of explained variance. While both models integrate individual importance, future studies should investigate which operationalization of individual importance is the most useful.

### Generalizability and limitations

The study aimed for a representative sample of survivors three years post‐stroke as they were continuously recruited in a prospective longitudinal study from a stroke unit, where >90% of stroke survivors are initially treated in hospitals in Germany (Kolominsky‐Rabas et al., 2006). An approximately equal sex ratio is expected in this population, as shown in our sample (Hillmann et al., 2017). We applied no age limit for participation, which led to the inclusion of *n* = 6 (5%) participants under the age of 50. This may be argued as less representative (Hillmann et al., 2017), although these survivors represent the reality of the recruiting stroke unit. Yet, participants showed a higher prevalence of history of depression (26–29%) compared with meta‐analytic evidence in stroke survivors (11%; Taylor‐Rowan et al., 2019), which limits the results' generalizability. The attrition from the study biased the sample in favour of participants with lower functional and cognitive impairment and younger age. Although this is a commonly observed phenomenon in longitudinal stroke studies (Towfighi et al., 2017), this may distinctly bias prevalence and variance of DS as functional and cognitive impairment are known determinants of post‐stroke depression (Ayerbe et al., 2013; de Ryck et al., 2014; Kutlubaev & Hackett, 2014). This may also explain the low prevalence of 18% for depressive symptoms above the cut‐off compared to 27% after one year or later in the literature (Liu et al., 2023). Cognitive impairment is also connected to reduced self‐awareness as a determinant of DS post‐stroke, which we did not control for (Wheeler et al., 2023). The selectivity may be even more attenuated by inclusion criteria on language and cognitive impairment at baseline. We applied these criteria to assure the validity of the outcome measures. The study called for completion of extensive self‐report measures at a very early stage a few days after stroke. Our experience from data collection confirmed these considerations. Many participants still needed individual assistance with outcome completion. However, this calls for the validation in older and, especially in regard to disability, cognition and language, more impaired populations.

As the most relevant limitation, the present study had a cross‐sectional design. This prevents direct conclusions for the predictivity of the described determinants. Most importantly, it cannot be concluded to what extent the reported effects are based on the negative perception bias in depression affecting the self‐report measures like self‐concept. However, the comparison of the unweighted and weighted associations of self‐concept subdomains with DS (Table 3) may argue for more complex relationships. The unweighted model showed no association of DS with subdomains, which are close to depression (Interested, Happy, Self‐confident, Satisfied). Weighting revealed some associations with these subdomains as well as the ones which are closer to functional independence (Capable, Independent, Active). If the associations are solely based on the negative perception bias in depression, the associations may be present also in the unweighted model.

Moreover, assessment of self‐concept and ‐discrepancy was retrospective, which aligns with previous studies (Ellis‐Hill & Horn, 2000; Lapadatu & Morris, 2019; Secrest & Zeller, 2007), yet this approach may limit reliability and is prone to memory bias. The ‘good‐old‐days’ bias requires consideration here (Gunstad & Suhr, 2001), which describes the tendency of individuals to see themselves in the past more positively. However, the individual experience of stroke was the focus of this study, which incorporates this psychological bias. Future studies should consider the aspect of self‐awareness as it is connected to mood and reasonable to affect the rating of one's self‐concept (Wheeler et al., 2023). Considering the evaluation of the IIWM assumptions, the restriction to three importance ratings resulted in ipsative data and hampered the application of a path analysis, which would have required an importance rating of all 18 self‐concept domains. This approach would be the most adequate statistical model to confirm the model's multivariate assumptions (Marsh & Scalas, 2018). However, the number of importance ratings was deliberately limited to margin participants' workload. We assumed that ordering 18 characteristics would be overwhelming and consequently invalid for stroke patients in acute ward. It must be noted that the use of the single HISDS items and their weighting were explorative analyses using the HISDS as the gold standard of self‐concept measures after brain injury. This approach requires further validation. Measures including self‐concept subdomains, which correspond with model assumptions (Shavelson et al., 1976) and show a sound factorial structure with a manageable number of factors, like the Tennessee Self‐Concept Scale‐2 (Fitts & Warren, 1996) could be applied in future studies. Moreover, models of global self‐concept are usually validated by using a measure of self‐esteem as a criterion (Scalas et al., 2013, 2017), which should be incorporated in future studies. Nevertheless, our findings certainly indicate to consider self‐concept, its subdomains and their individual relevance in future, especially longitudinal, research. These studies should also cover the time point of 6 months after stroke, which was reported to be crucial in the development of post‐stroke depression (Werheid, 2016).

## CONCLUSION

The findings of the present study highlight the role of self‐concept in post‐stroke depression and hence the need to further examine this relationship. Special attention should be paid to the temporal changes in self‐perception related to brain injury and depression, respectively, the longitudinal predictive value of global self‐concept, and the influence and therapeutic use of individual importance‐weighting of self‐concept subdomains. In accordance with these and previous findings (Duits et al., 2008; Kusec & Demeyere, 2024), subjective beliefs about stroke sequelae should be the scope of future post‐stroke depression research. Finally, clinical research and practice may focus on the development, evaluation and application of psychological interventions, which include self‐concept as a target variable to tackle the burden of depression after stroke.

## AUTHOR CONTRIBUTIONS

**Simon Ladwig:** Conceptualization; investigation; methodology; writing – original draft; formal analysis. **Katja Werheid:** Conceptualization; writing – review and editing; methodology; supervision.

## CONFLICT OF INTEREST STATEMENT

The authors have no financial support or conflicts of interest to disclose, which may have affected this study.

## Data Availability

Original data are available under the following link: https://data.mendeley.com/datasets/mcfmw5zgr5/1.
